# Contrast sensitivity and retinal straylight after alcohol consumption: effects on driving performance

**DOI:** 10.1038/s41598-020-70645-3

**Published:** 2020-08-12

**Authors:** Miriam Casares-López, José J. Castro-Torres, Francesco Martino, Sonia Ortiz-Peregrina, Carolina Ortiz, Rosario G. Anera

**Affiliations:** Laboratory of Vision Sciences and Applications, Department of Optics, Facultad de Ciencias (Edificio Mecenas), University of Granada, Avenida Fuentenueva s/n, 18071 Granada, Spain

**Keywords:** Health care, Risk factors

## Abstract

In this study, we aimed to investigate the effects of alcohol intake on visual function and driving performance, as well as on the relationship between these. A total of 40 healthy participants took part in three experimental sessions: one baseline session and two further sessions after consuming two different quantities of alcohol (300 ml and 450 ml of red wine). The breath alcohol content (BrAC) was measured using a breath analyzer. The contrast sensitivity and retinal straylight due to the forward intraocular scattering were measured to characterize visual function, and driving performance was assessed in three different scenarios using a driving simulator. The results showed a deterioration in contrast sensitivity and retinal straylight after drinking alcohol, in addition to an impaired ability to drive, especially for the highest alcohol intake. We also observed that the deteriorated driving performance was a function of the contrast sensitivity and retinal straylight under the effects of alcohol, indicating that these visual variables can partially predict driving performance in these conditions.

## Introduction

Vision plays an important role in driving, in such a way that impaired vision has negative effects on driving performance^1,2^. For this reason, minimum visual requirements must be met to obtain or renew a driving license and, although these requirements may vary according to the country, they generally include visual acuity and visual field^3^. However, there is evidence indicating that other visual variables also significantly influence driving^1,4,5^. In this sense, some studies have revealed the importance of visual functions such as contrast sensitivity and glare sensitivity in driving performance^4,6,7^. In particular, it has been suggested that retinal straylight is valuable for assessing visual function in drivers^6,8^. Straylight is defined as the skirt of retinal light distribution from a point source of light^9^, and has been widely used as an indicator of glare sensitivity^6,10–12^. It is intimately related to intraocular scattering and glare, although these are not exactly the same. The straylight effect is caused by the intraocular scattered light reaching the retina, causing a deterioration in image contrast^12^. This association between straylight and contrast sensitivity has been widely studied. Puell and Palomo-Álvarez observed a deterioration in contrast sensitivity in the presence of forward light scatter^11,13^, and Patterson and colleagues concluded that light scatter on the retina acts as a predictor of contrast sensitivity, demonstrating that these two variables are closely related^14^.

With regard to driving performance, impaired contrast sensitivity has been related to increased crash risk^15^. In addition, some authors have shown that drivers who reported greater glare, also had higher straylight^6,8^.

In addition to visual quality, another major factor affecting normal driving performance is alcohol consumption, which is considered to be one of the major global public health problems, accounting for more than 5% of all deaths each year^16^. This legal drug also impairs visual performance, including contrast sensitivity. Although it is clear that low to moderate alcohol consumption produces a transient deterioration in contrast sensitivity^17,18^, there is no agreement as to which spatial frequencies are most affected by alcohol. Likewise, it is known that in low to moderate doses alcohol increases intraocular scattering and the perception of halos^19^. However, even though intraocular scattering is closely related to straylight, as mentioned, and halo perception could also be related, there is no information on the effects of alcohol use on retinal straylight, and its influence on the ability to drive.

Considering that driving involves an important public safety component, the study of DUI (Driving Under the Influence) behavior is of particular interest. In fact, it has been estimated that alcohol-related accidents are responsible for between 27 and 30% of all traffic fatalities in Spain and the US^20,21^, and even low doses of alcohol increase the risk of having a road accident^16^. Numerous authors have revealed that alcohol consumption impairs driving performance. Some aspects, such as steering behavior^21^, standard deviation of the lateral position (SDLP)^22,23^ and hazard perception^24^ are negatively affected following alcohol use, especially for moderate and high doses. Likewise, various authors agree that DUI is also connected with an increased crash risk^25,26^. However, the results on how alcohol affects mean speed are controversial.

As the association between driving performance and vision has already been established, we hypothesize that there is also a relationship between deteriorated driving and visual performance under the effects of alcohol. The purpose of this study is, therefore, to analyze contrast sensitivity and retinal straylight, as well as their involvement in driving performance following the consumption of two different doses of alcohol.

## Methods

### Participants

All the methods used and described in this paper were approved by the University of Granada Human Research Ethics Committee (921/CEIH/2019). A total of 40 participants (18 females and 22 males) ranging in age from 20 to 56 years (average 28.4 ± 10.4 years), completed the experiment. Before starting the experiment, they all signed an informed consent form in accordance with the Helsinki Declaration. The informed consent included information on the purpose of the study and methods used, as well as the amount of alcohol they had to drink in each session. The inclusion criteria were: monocular visual acuity ≥ 1.0 (decimal notation) with best correction, no ocular diseases, no pathological conditions that could be affected by alcohol intake, having had a valid driving license for at least one year, and being a social drinker. To rule out alcohol-use related disorders, the participants took the Alcohol Use Disorders Identification Test (AUDIT) and had a score of 8 or less, indicating that they were not suffering alcohol dependence.

### Alcohol administration

To create an environment as similar as possible to real-life social drinking, we used wine^27,28^. The wine was red wine (Pago de Almaraes wineries, S.L. Benalúa de Guadix, Granada, Spain) with a 13.5% alcohol content. The participants were able to drink over a 40 min period. At the two sessions, they were given different doses: 300 ml to represent a low-moderate alcohol intake (after Alcohol Consumption 1, aAC1); and 450 ml to represent a moderate-high alcohol intake (after Alcohol Consumption 2, aAC2). Thirteen minutes after finishing the dose, their breath alcohol content (BrAC) was measured using the Dräger Alcotest 6,810 breath analyzer (Dräger Safety AG & Co. Lübeck, Germany), and this was remeasured every 20 min. If necessary, a second dose was provided during the session in order to maintain the BrAC level ± 0.05 mg/l. The final BrAC level was the mean value of the four measurements taken over the session. According to the average BrAC reached in the last session (aAC2), the participants were assigned to two groups: BrAC ≤ 0.25 mg/l (low BrAC, n = 15) and BrAC > 0.25 mg/l (high BrAC, n = 25), as 0.25 mg/l is the legal limit for driving in Spain and the most common legal BrAC worldwide (51 countries)^16^.

### Visual performance

Contrast sensitivity (CS) was measured using the VistaVision contrast sensitivity test (DMD MedTech, Villarbasse, Torino, Italy), which comprises sinusoidal grids for which the observers had to recognize and indicate whether the grid inclination was to the right, the left, or vertical. Eight different contrast sensitivity levels were assessed in this test, at six different spatial frequencies: 0.75, 1.5, 3, 6, 12 and 18 cycles per degree (cpd) of visual angle.

Binocular and monocular measurements were made at 3 m. The test had a background luminance of 60 cd/m^2^ and was performed in mesopic lighting conditions. For the monocular assessment, one eye was randomly selected, as recommended in other work^29^.

The forward scattering affecting the retinal image was tested by measuring the retinal straylight. To do this, we used the OCULUS C-Quant straylight meter (Oculus DG, Germany), whose reliability has been widely proved in research^6,30^. The test consists of an outer ring, which represents the straylight source and presents a real flicker, and the central test, divided into two fields. One of these two central fields does not flicker, but due to the scattered light reaching the retina, it may be perceived as flickering. The device uses the compensation comparison method, where observers have to decide which of the two fields is flickering more intensely. The test is performed monocularly. At the end of the test, the logarithm of the retinal straylight (log[s]) is given, which may vary according to age. Thus, the older the observer is, the higher the normal log(s) value^10^. For each subject, the eye selected for the straylight measurement was the same one as randomly selected for the monocular CS. This test was also performed in dim surroundings.

### Driving performance

A driving simulator was used to evaluate driving performance, as these have been demonstrated to correlate well with real driving^31^. The software used was the Simax driving simulator v4.0.8 beta (SimaxVirt S.L., Pamplona, Spain). A virtual environment was presented in three 27-inch high-definition screens. The simulator consists of a car seat, a steering wheel with a maximum rotation of 900 degrees, and a six-speed gearshift with reverse, and three pedals: clutch, accelerator, and brake (Logitech International S.A., Lausanne, Switzerland)^4,32^.

None of the participants were affected by simulator sickness and, therefore, none were discarded for this reason. Most of the participants were young and they had become accustomed to the simulator in the previous training sessions.

Every participant drove along a 12.5 km itinerary consisting of three different sections: first, a dual carriageway Section (4.5 km); then a mountain road section of one-lane single carriageway (6.0 km); and, finally, an inner-city circuit (2.0 km). Different driving variables were analyzed in each section. The speed was assessed to provide the mean speed (km/h) and the standard deviation of the speed was calculated (SDSP, km/h). The position of the car on the road was assessed to give the standard deviation of the lateral position (SDLP, m), the distance driven on the shoulder (m), and the number of times the driver veered onto the shoulder (NTVOS). To evaluate the steering behavior, the standard deviation of the angular velocity of the steering wheel (SDωSW, rad/s) was calculated, and, finally, the reaction time (s) was provided. Reaction time was assessed using the braking responses generated by the driving simulator along the mountain road, when the car driving ahead braked suddenly. The response time was therefore calculated as the interval between the instant the brake lights turned on in the preceding car and when the driver in the simulator pressed the brake pedal. Collisions were not considered, as it has been suggested that an analysis of crashes in simulated driving does not faithfully represent real driving performance^33^. An overall driving performance score (ODPS) was calculated by averaging the z-scores of the driving variables included in the study in the three sections (mean speed, SDSP, SDLP, NTVOS, distance driven onto the shoulder, SDωSW, and reaction time), and equal weighting was assigned to all variables, in line with other studies^32,34^. As higher values of these driving variables indicate worse driving performance, we multiplied the z-scores by − 1 to obtain a driving score that was comparable with previous studies. In this way, lower ODPS values indicated worse driving performance.

### Experimental sessions

The participants took part in five sessions, each one week from the next. There were two training sessions to learn how to use the driving simulator and how to perform the visual tests properly, and then three further sessions that were compared and analyzed: one baseline (baseline session) and two after consuming alcohol (aAC1 and aAC2). Visual performance was analyzed under both binocular conditions (whenever possible) and monocular conditions (by randomly selecting one eye). All the visual and driving tests were performed under these conditions (baseline and two after drinking alcohol). The measurements were taken in the Laboratory of Vision Sciences and Applications at the University of Granada.

### Data analysis

We used SPSS Statistics v.20 software (SPSS Inc., Chicago, IL, U.S.) for the statistical analysis. The mean values ± standard errors were reported for all the variables analyzed. The Kolmogorov–Smirnov test was used to examine the normality of the residuals.

An ANOVA test for repeated measures was conducted to analyze the effect of alcohol intake on normal visual and driving performance data for each dependent variable, providing the F statistic, p-value and effect size (η_p_^2^) with pairwise comparisons using Bonferroni correction to analyze the differences between the experimental sessions. A Friedman test using a two-way ANOVA was conducted to analyze the differences between sessions for non-normal data, providing the χ^2^ statistic and p-value.

Finally, a multiple linear regression analysis and Spearman test was run to analyze the relationship between vision and driving performance, providing the correlation index (ρ) and the p-value. A significance level of 95% was considered for all the tests.

## Results

### BrAC level

**Figure 1:**
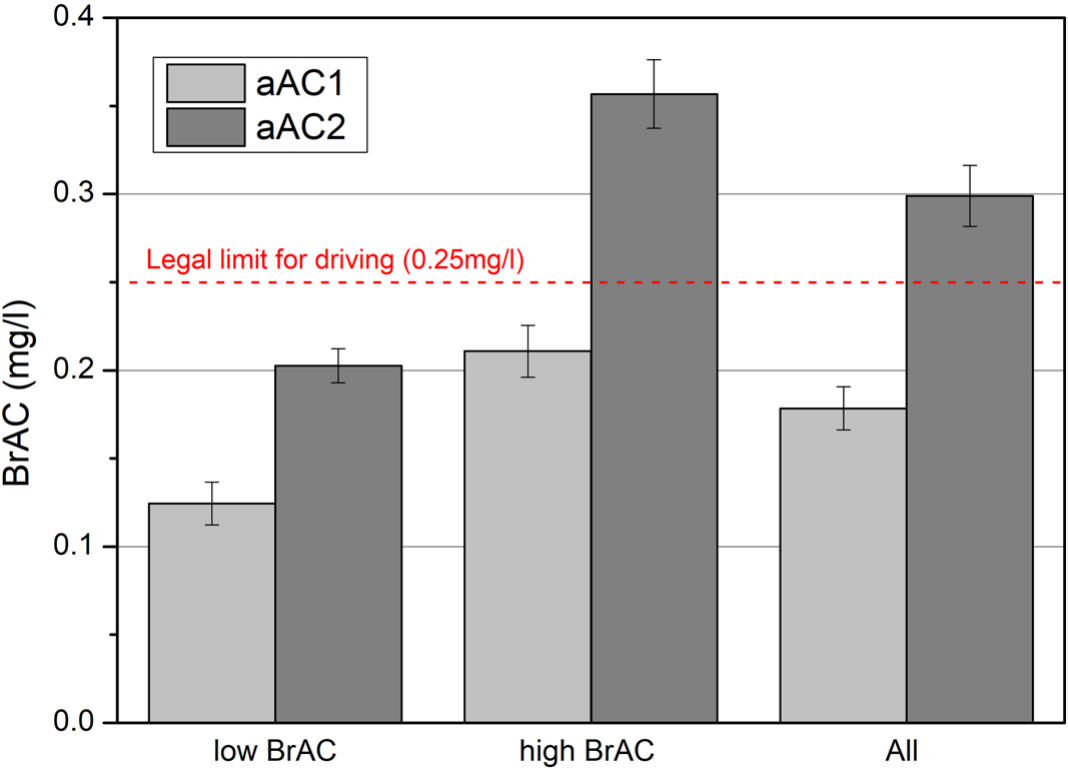
Mean BrAC level for the two groups (low and high BrAC) and combined in the aAC1 and aAC2 sessions. The red line indicates the legal limit for driving (0.25 mg/l) in several countries. Standard errors included.

The participants reached a mean BrAC level of 0.18 ± 0.01 mg/l after drinking 300 ml of wine (aAC1 condition), and 0.30 ± 0.02 mg/l after 450 ml (aAC2 condition). Only 17.5% reached the legal limit for driving (0.25 mg/l) in the aAC1 session, and 62.5% (the high BrAC group) reached that limit in the aAC2 session. The participants were assigned to two groups according to the BrAC level they attained in the aAC2 condition (Fig. 1). None of the participants in the low BrAC group reached the legal limit in either the aAC1 session (mean 0.12 ± 0.01 mg/l), or the aAC2 session (mean 0.20 ± 0.01 mg/l). All the participants in the high BrAC group reached a BrAC greater than 0.25 mg/l in the aAC2 session (mean of 0.36 ± 0.02 mg/l). In this group, 43.75% of the participants exceeded the legal limit for driving in the aAC1 session (mean of 0.21 ± 0.01 mg/l).

### Visual performance

Table 1 presents the mean contrast sensitivity (monocular and binocular). For each subject and session, the contrast sensitivity (CS) was taken as the average of all the spatial frequencies assessed. For both groups, the mean CS deteriorated after consuming alcohol, especially in the high BrAC group in the aAC2 condition.

**Table 1:** Mean monocular (MON) and binocular (BIN) CS for the two BrAC groups (low BrAC and high BrAC) and all participants in the three sessions: baseline, aAC1, and aAC2.

Contrast sensitivity (CS)				
		Baseline	aAC1	aAC2
Low BrAC	MON	121.7 ± 5.7	105.0 ± 6.7	98.4 ± 5.6
	BIN	147.6 ± 4.4	137.4 ± 5.5	140.1 ± 5.5
High BrAC	MON	125.2 ± 3.3	109.0 ± 3.5	101.8 ± 3.7
	BIN	153.7 ± 2.6	140.3 ± 4.2	135.7 ± 4.6
All	MON	125.5 ± 3.3	107.6 ± 3.3	101.3 ± 3.4
	BIN	151.3 ± 2.3	139.6 ± 3.2	137.2 ± 3.5

Standard errors included.

Monocular CS was significantly affected by alcohol in the low BrAC group at the spatial frequencies of 6 cpd [χ^2^(2) = 11.056; p = 0.004], 12 cpd [χ^2^(2) = 9.000; p = 0.003] and 18 cpd [χ^2^(2) = 7.515; p = 0.023]. In the high BrAC group, monocular CS significantly deteriorated at the spatial frequencies of 0.75 cpd [χ^2^(2) = 13.087; p < 0.001], 1.5 cpd [χ^2^(2) = 10.903; p = 0.004], 3 cpd [χ^2^(2) = 7.032; p = 0.030], 6 cpd [χ^2^(2) = 11.207; p = 0.004] and 12 cpd [χ^2^(2) = 6.864; p = 0.032]. Binocular CS deteriorated in the high BrAC group at 6 cpd [χ^2^(2) = 15.292; p < 0.001] and 12 cpd [χ^2^(2) = 10.618; p = 0.005], but only for 12 cpd in the low BrAC group [χ^2^(2) = 7.357; p = 0.025].

**Figure 2:**
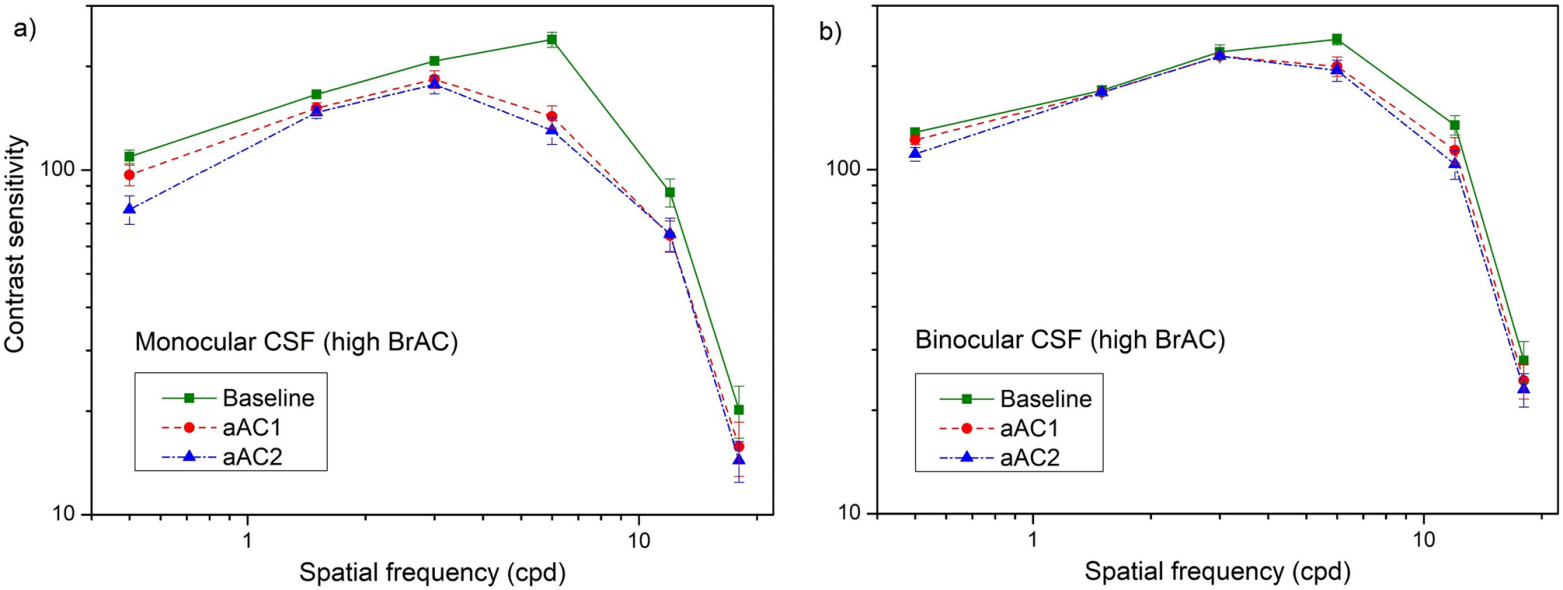
(**a**) Monocular and (**b**) binocular CS for the high BrAC group in the three experimental sessions (baseline, aAC1, and aAC2). Standard errors included.

As shown in Fig. 2a,b, the contrast sensitivity in the high BrAC group was lower after consuming alcohol in the aAC1 and aAC2 experimental sessions, especially at the spatial frequency of 6 cpd. On average, the highest logCS difference was reached monocularly for the spatial frequency of 6 cpd comparing the baseline condition with the aAC2 condition, attaining a difference of 0.26 (Fig. 2a), which is above the clinically significant difference of 0.1; this also occurs at higher spatial frequencies (12 and 18 cpd). A similar but less well-defined tendency was found for the baseline-aAC1 comparison, where the highest logCS difference was again reached at 6 cpd. With this in mind, the CS deterioration was higher monocularly, demonstrating the superiority of the binocular system. Statistically, the differences between the sessions revealed significant deterioration between the baseline and aAC2 monocular CS for 0.75 cpd (p = 0.042) and 6 cpd (p = 0.035). In the binocular CS, significant deterioration was found between the baseline and aAC2 condition for 6 cpd (p = 0.013) and 12 cpd (p = 0.042), corroborated with a clinically significant deterioration for these spatial frequencies.

In the low BrAC group, the pairwise comparisons showed significant deterioration in the monocular CS for 6 cpd (p = 0.032) and 12 cpd (p = 0.008) comparing the baseline and aAC2 sessions, but no significant differences were found in the binocular CS. No significant differences were observed between the baseline and aAC1 conditions and between the aAC1 and aAC2 conditions in either of the groups.

**Figure 3:**
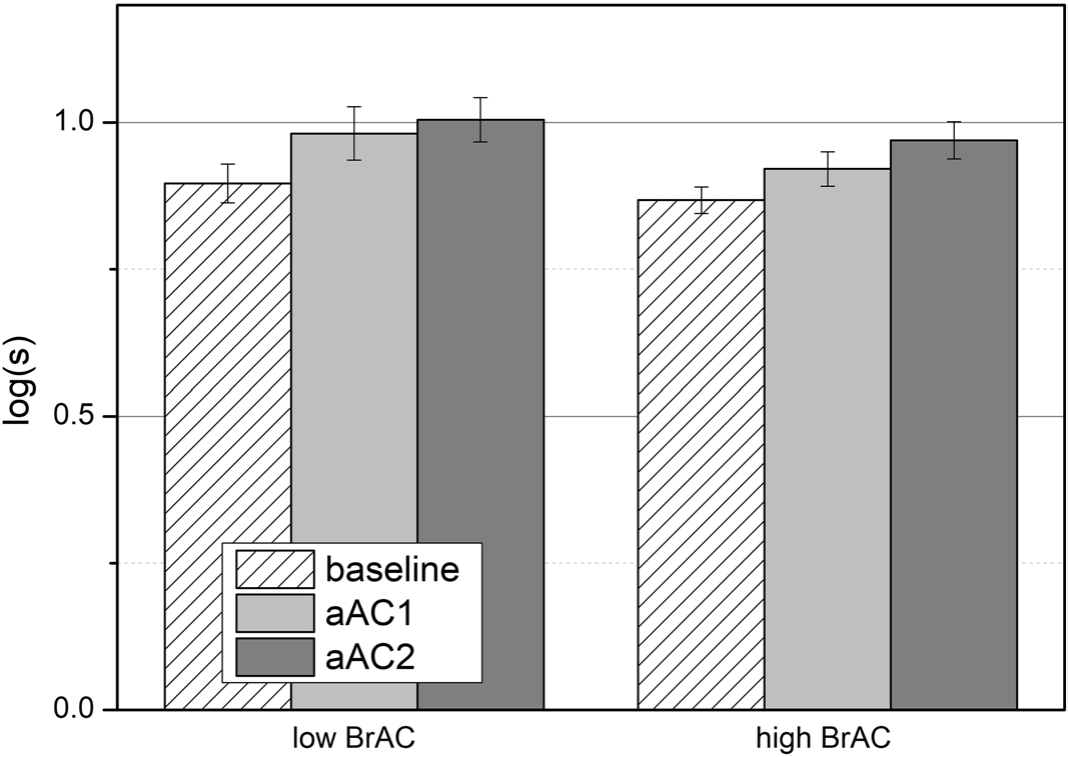
Mean log(s) values for the three experimental sessions (baseline, aAC1, and aAC2) the two BrAC groups (low and high BrAC). Standard errors included.

Mean log(s) values are represented in Fig. 3. In the low BrAC level group, the mean values were 0.90 ± 0.03 in the baseline session, 0.98 ± 0.05 in the aAC1 session and 1.00 ± 0.04 in the aAC2 session; the mean values in the high BrAC group were 0.87 ± 0.02 in the baseline session, 0.92 ± 0.03 in the aAC1 session and 0.97 ± 0.03 in the aAC2 session. After alcohol consumption, the retinal straylight was significantly higher in the low BrAC group [F(2,14) = 25.055; p < 0.001; η_p_^2^ = 0.996], as well as in the high BrAC group [F(2,24) = 24.835; p < 0.001; η_p_^2^ = 0.997]. Pairwise comparisons showed a significant deterioration of the log(s) in the aAC1 condition with respect to the baseline condition for both the low BrAC (p = 0.036) and high BrAC (p = 0.010) groups; the same was found for the aAC2 condition in both groups: low BrAC (p = 0.001) and high BrAC (p < 0.001).

### Driving performance

#### Dual Carriageway

The results of the driving variables on the dual carriageway are presented in Table 2. As shown in the table, the SDLP, the NTVOS and SDωSW were, on average, higher after consuming alcohol. Only the SDωSW in the high BrAC group was significantly deteriorated [χ^2^(2) = 22.160; p < 0.001]. Analyzing the differences between the sessions, we found a significant difference in the SDωSW between the baseline and aAC2 sessions (p < 0.001) as well as between the aAC1 and the aAC2 sessions (p < 0.001) in this group. No significant differences were observed in either mean speed or SDSP in the low BrAC group, and although a slight increase in these variables was observed in the high BrAC group, the difference was not significant.

**Table 2:** Mean values of driving variables on the dual carriageway and in the inner-city for the two groups (low BrAC and high BrAC) in the three experimental sessions (baseline, aAC1, and aAC2).

Group		Mean speed (Km/h)	SDSP (km/h)	SDLP (m)	NTVOS	SDωSW (rad/s)
**Dual carriageway**						
Low BrAC	Baseline	116.66 ± 1.71	9.06 ± 0.92	0.55 ± 0.05	4.00 ± 0.80	0.16 ± 0.02
	aAC1	116.60 ± 1.75	8.09 ± 0.84	0.57 ± 0.05	4.20 ± 0.87	0.17 ± 0.02
	aAC2	115.32 ± 1.73	8.64 ± 0.85	0.59 ± 0.04	4.53 ± 0.79	0.20 ± 0.03
High BrAC	Baseline	117.35 ± 1.59	9.63 ± 0.56	0.54 ± 0.02	3.12 ± 0.46	0.23 ± 0.02
	aAC1	119.62 ± 1.71	9.96 ± 0.92	0.57 ± 0.03	4.63 ± 0.80	0.24 ± 0.02
	aAC2	119.08 ± 1.86	12.08 ± 1.77	0.63 ± 0.04	5.92 ± 1.11	0.38 ± 0.06
**Inner-city**						
Low BrAC	Baseline	33.55 ± 2.25	19.14 ± 0.94	0.87 ± 0.08	14.31 ± 1.09	1.05 ± 0.08
	aAC1	33.84 ± 1.63	18.84 ± 0.85	0.93 ± 0.07	15.46 ± 1.13	1.09 ± 0.05
	aAC2	33.39 ± 1.36	19.63 ± 0.85	0.96 ± 0.09	14.77 ± 1.09	1.17 ± 0.06
High BrAC	Baseline	32.05 ± 1.13	18.08 ± 0.76	0.95 ± 0.06	14.20 ± 0.57	1.17 ± 0.05
	aAC1	32.00 ± 1.27	19.13 ± 0.97	0.96 ± 0.07	15.08 ± 1.01	1.21 ± 0.07
	aAC2	33.33 ± 1.47	20.52 ± 1.16	1.02 ± 0.07	17.08 ± 1.00	1.45 ± 0.10

Standard errors included.

#### Two-lane mountain road

Table 3 presents the results of the driving variables on the mountain road. In the high BrAC group, alcohol consumption significantly affected most of the driving parameters: SDSP [F(2,24) = 7.927; p = 0.010; η_p_^2^ = 0.769], SDLP [χ^2^(2) = 11.217; p = 0.004], distance driven on the shoulder [χ^2^(2) = 17.711; p < 0.001], NTVOS [χ^2^(2) = 24.364; p < 0.001], SDωSW [F(2,24) = 11.905; p = 0.002; η_p_^2^ = 0.332], and reaction time [F(2,24) = 9.410; p = 0.006; η_p_^2^ = 0.309]. In the low BrAC group, the alcohol intake had a significantly negative effect on the SDLP [F(2,14) = 8.183; p = 0.015; η_p_^2^ = 0.427], the NTVOS [χ^2^(2) = 17.348; p < 0.001], and SDωSW [χ^2^(2) = 7.000; p = 0.030]. No significant differences were observed in the mean speed for either group.

**Table 3:** Mean values of driving variables on the two-lane mountain road for the two groups (low and high BrAC) in the three experimental sessions (baseline, aAC1, and aAC2).

Group		Mean speed (Km/h)	SDSP (Km/h)	SDLP (m)	Distance driven on the shoulder (m)	NTVOS	SDωSW (rad/s)	Reaction time (s)
**Two-lane mountain road**								
Low BrAC	Baseline	56.44 ± 0.59	21.43 ± 0.77	0.51 ± 0.17	49.74 ± 24.19	1.54 ± 0.42	0.57 ± 0.02	0.84 ± 0.05
	aAC1	55.79 ± 0.41	20.45 ± 0.59	0.53 ± 0.02	62.32 ± 25.71	2.23 ± 0.60	0.59 ± 0.03	0.86 ± 0.04
	aAC2	56.11 ± 0.37	20.79 ± 0.47	0.60 ± 0.03	53.69 ± 15.86	4.23 ± 0.81	0.62 ± 0.02	0.93 ± 0.05
High BrAC	Baseline	55.08 ± 0.37	21.78 ± 0.50	0.60 ± 0.03	90.00 ± 33.27	5.12 ± 1.16	0.70 ± 0.03	0.89 ± 0.03
	aAC1	56.10 ± 0.47	21.81 ± 0.60	0.60 ± 0.03	104.75 ± 26.94	6.04 ± 1.04	0.73 ± 0.03	1.00 ± 0.04
	aAC2	54.39 ± 1.35	23.76 ± 0.87	0.74 ± 0.05	297.85 ± 103.80	11.08 ± 2.08	0.90 ± 0.06	1.04 ± 0.04

Standard errors included.

Pairwise comparisons in the high BrAC group showed a significant increase in the SDSP in the aAC2 session compared to the baseline (p = 0.029). The SDLP was also significantly higher in the aAC2 session with respect to the baseline condition (p = 0.002), and there was also a significant difference between the aAC1 and the aAC2 sessions (p = 0.003). The distance driven on the shoulder and the NTVOS were significantly higher when comparing the baseline and aAC2 sessions (p < 0.001 for both variables) and the aAC1 and aAC2 sessions (p = 0.002 and p = 0.001, respectively, for the variables). The SDωSW was significantly higher after alcohol consumption when comparing the baseline and aAC2 sessions (p = 0.006), and reaction time was also higher in the aAC1 and aAC2 conditions compared with the baseline session (p = 0.010 and p = 0.004, respectively).

In the low BrAC group, significant differences were found between the baseline and aAC2 sessions for the SDLP (p = 0.046) and SDωSW (p = 0.011). The NTVOS was also significantly higher in the aAC2 condition with respect to both the baseline condition (p = 0.006) and the aAC1 condition (p = 0.015).

#### Inner-city

The inner-city driving results are given in Table 2. In the high BrAC group, alcohol intake had a significant effect on the NTVOS [χ^2^(2) = 9.692; p = 0.008], as well as the SDωSW [χ^2^(2) = 7.535; p = 0.023]. Although the SDLP and SDωSW were higher for the low BrAC group after consuming alcohol, these differences were not significant. The mean speed was not affected by alcohol consumption in either group. However, the SDSP was higher, on average, in the high BrAC group under the effects of alcohol, although not significantly.

Pairwise comparisons in the high BrAC group showed that the NTVOS in the aAC2 condition was significantly higher compared with the baseline condition (p = 0.006). The same was observed for the SDωSW when comparing the aAC2 condition with both the baseline (p < 0.001) and aAC1 (p = 0.032) conditions.

### Driving score

**Figure 4:**
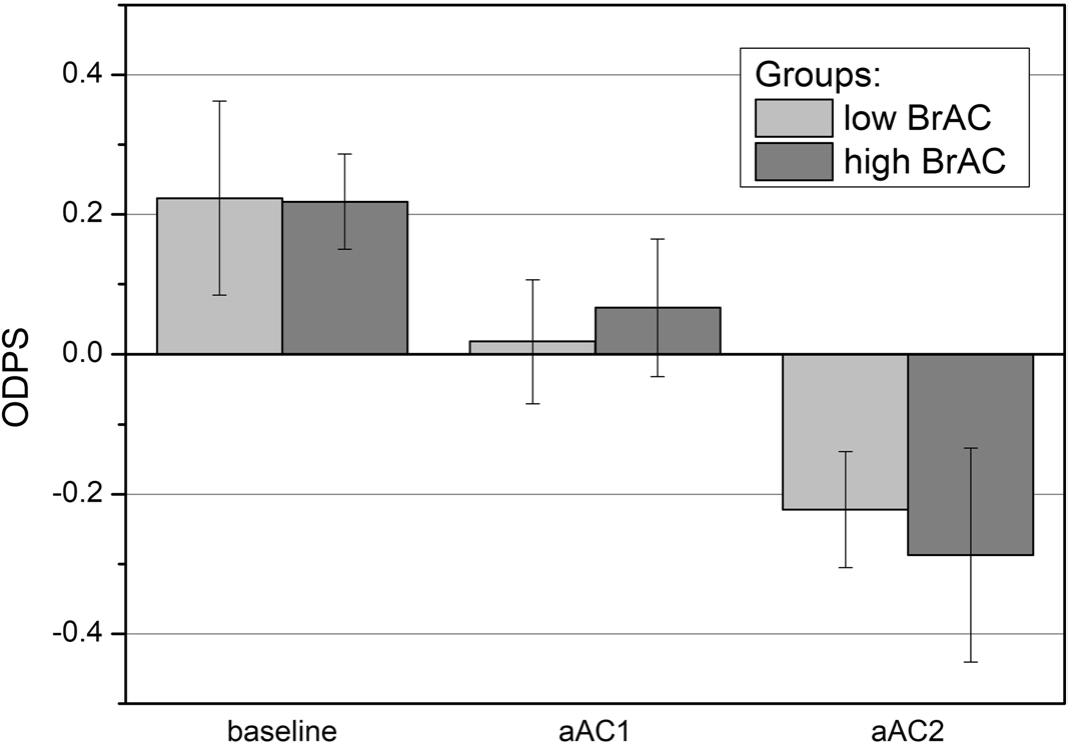
Mean driving scores in the three experimental sessions (baseline, aAC1, and aAC2) for the low and high BrAC groups. Standard errors included.

The mean ODPS values for the low BrAC and high BrAC groups, respectively, were 0.22 ± 0.14 and 0.22 ± 0.07 in the baseline session, 0.02 ± 0.09 and 0.07 ± 0.10 in the aAC1 session, and − 0.22 ± 0.08 and − 0.29 ± 0.15 in the aAC2 session (Fig. 4). The ODPS was significantly affected by alcohol consumption in both the low BrAC [F(2,14) = 23.944; p < 0.001 η_p_^2^ = 0.631] and high BrAC [F(2,24) = 12.979; p = 0.001; η_p_^2^ = 0.351] groups. Pairwise comparisons showed a significant deterioration of the ODPS in the aAC2 session compared to the baseline session for the low BrAC and high BrAC groups (p = 0.001 and p = 0.004, respectively). The differences were also significant between the aAC1 and aAC2 sessions (p = 0.034) for the high BrAC group.

### Relationship between vision and driving performance

**Figure 5:**
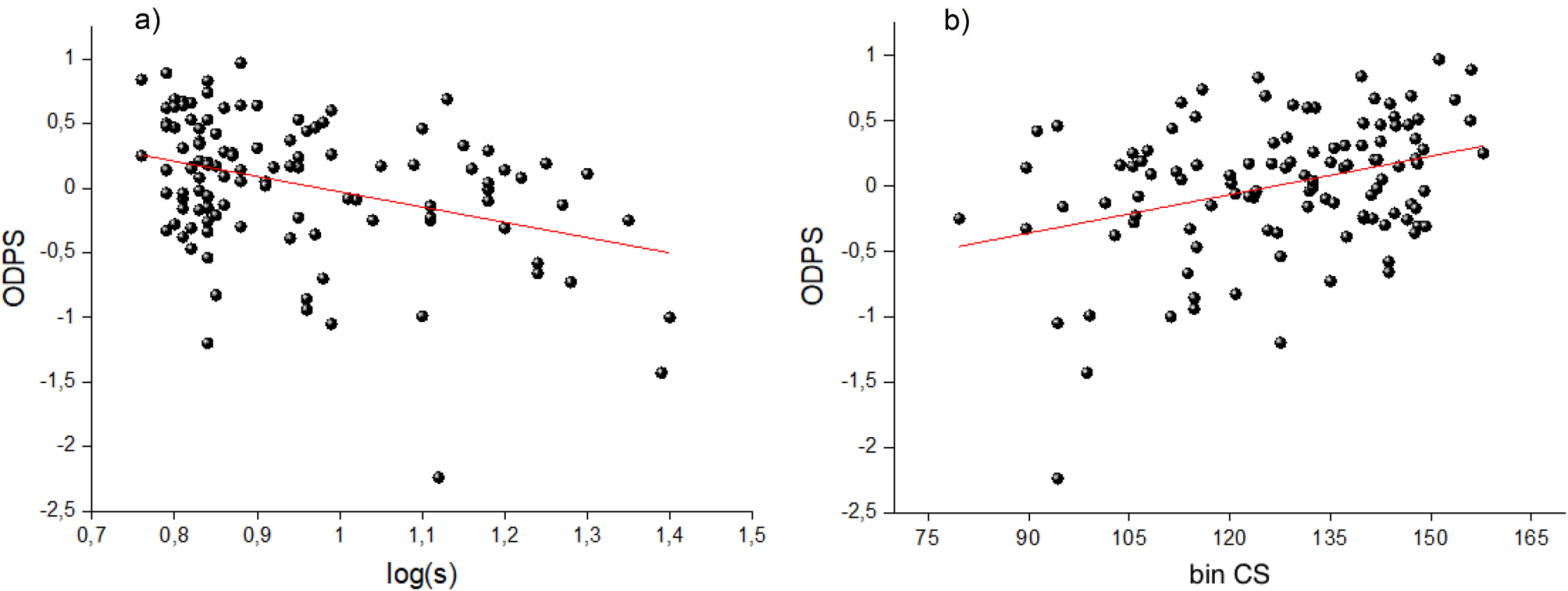
The ODPS as a function of (**a**) log(s), and (**b**) the binocular contrast sensitivity (binCS).

The strongest correlation between vision and driving performance (ODPS) under the effect of alcohol was found for retinal straylight (r = − 0.350; p < 0.001) and the binocular CS (r = 0.251; p = 0.006), as shown in Fig. 5a,b. To find the best linear model to predict driving score using the visual parameters, a multiple lineal regression analysis was performed with the ODPS as the dependent variable and the different visual tasks as independent variables. The variables included in the model were chosen using a forward selection. The visual parameters included were the binocular CS (binCS) and retinal straylight (log[s]), which explained 16.1% of the driving performance under these conditions (r = 0.401; p = 0.025). The ODPS would be given by the following regression line (Eq. 1):

1 $$ ODPS = - 1.146 \times \log \left( s \right) + 0.006 \times binCS + 0.248. $$

The standard deviations were 0.346, 0.003 and 0.567, for the first, second and third terms of the equation, respectively.

## Discussion

Our results show deteriorated visual performance after consuming alcohol through reduced contrast sensitivity and increased retinal straylight. According to the International Council of Ophthalmology^35^, contrast sensitivity and glare sensitivity should be included within the visual test for obtaining a driving license. In our study, deterioration in the monocular and binocular CS was observed in both groups, low BrAC and high BrAC, particularly in the latter for the aAC2 condition, i.e., when the BrAC level was above the legal limit for driving (0.25 mg/l). This deterioration was especially important for the spatial frequencies of 6 cpd and 12 cpd. Most studies on the effects of alcohol on CS show deterioration after using alcohol when considering stationary targets^17,18,36^, especially at high doses^37^. However, it is not clear whether all spatial frequencies are equally affected by alcohol use; some works have shown that low and high spatial frequencies are the most affected^36,37^. Other authors maintain that all spatial frequencies are equally affected^38,39^. The amount of alcohol ingested and the test used to assess the CS may be responsible for this disparity. Our results are in accordance with findings indicating that the medium–high spatial frequencies are the most impaired by alcohol. Since it has been suggested that deteriorated contrast sensitivity to low spatial frequencies is attributed to a toxic effect of alcohol on the optic nerve^40^, it seems that this nerve may not be significantly affected by the doses provided in this study. Zhuang and colleagues also suggested that the increased threshold observed for high spatial frequencies may be due to alcohol interfering with the parvocellular pathway, which may be the main cause of impaired contrast sensitivity^40^.

On the other hand, increased retinal straylight (log[s]) has also been observed following alcohol consumption. This increase in the log(s) was similar in both groups, low BrAC and high BrAC. However, the differences between the aAC1 and aAC2 conditions were greater in the high BrAC group, where greater deterioration in the log(s) was observed for BrAC levels over the legal limit. To the authors’ knowledge, there are no previous reports on the effects of alcohol on retinal straylight, but there is evidence of how alcohol influences intraocular scattering. Some authors have observed increased intraocular scattering and deteriorated retinal image quality after alcohol use^19,41^. Alcohol has been detected in the tear film, leading to a decrease in tear breakup time (TBUT)^42^. This change in tear film composition has been suggested to be one of the main factors affecting intraocular scattering and retinal image quality following alcohol consumption, along with the increased pupil size observed under these conditions^19,43^. As intraocular scattering and retinal straylight are closely related, an increase in the opacities and irregularities of the ocular media leads to increased retinal straylight^44^. Retinal straylight evaluates the forward scattered light in the retina, measured for wide visual angles (5 to 10 degrees)^10^. The objective scatter index (OSI), a parameter which also evaluates intraocular scattering, corresponds to the ratio between the peak of the double-pass image and a ring between the 12 and 20 min arcs around the central peak^45^. Therefore, retinal straylight is measured for large visual angles compared with the angles analyzed using the double-pass image of the OSI parameter. Although both parameters are substantially different, a similar tendency can be expected when strongly increasing forward intraocular scattering in various conditions. However, the different methods used (psychometric versus objective measurement; large angles versus small angles) should be taken into account as these could result in different outcomes in some experimental conditions. Measuring intraocular scattering is of particular interest for clinical applications, such as cataract classification, since it objectively measures the amount of light scattered through the ocular media. However, the straylight measurement provides information closer to the visual perception of the subject, by means of the compensation comparison method, taking into consideration the effects of the forward scattering on the retinal image. In this sense, as reported in other work, it would be interesting to evaluate the role of retinal straylight in visual tasks such as driving^6,8^.

For driving performance, the driving variables analyzed in the three scenarios were found to be deteriorated after alcohol consumption. This reduction was the greatest in the high BrAC group, especially in the aAC2 condition. Likewise, the overall driving performance score (ODPS) was significantly reduced under the effects of alcohol, especially when the BrAC level was above the legal limit (Fig. 4). The environment and road layout both importantly influence driving performance^46^, in such a way that the more complex the scenario is, the more likely this is to be impaired by alcohol^22,33^. In this sense, our results showed that the two-lane mountain road was the section of the route where the driving variables presented the greatest deterioration, as there are more curves, more changing speed-limits and more traffic. In this scenario, we observed a significant impairment of most of the driving variables after alcohol use, especially for the high alcohol intake. We also observed a significant deterioration in driving parameters in the inner city. However, on the dual carriageway, only the SDωSW was significantly different after alcohol use. Mean speed was the only variable that did not change in a noteworthy way between the three experimental sessions; a slight increase in mean speed was observed on the dual carriageway for the high BrAC group following alcohol consumption, but this was not significant. There is no agreement in the literature on whether mean speed is affected by alcohol consumption or not. Some authors have concluded that this parameter remains unaltered under the influence of alcohol in low-moderate doses^24,47^, in line with our findings. On the other hand, some authors have reported increased speed as drivers adopt risky behavior^48,49^, and yet others have observed that, under the effects of alcohol, drivers go slower in an attempt to compensate for a situation that seems difficult^33^. However, our results did show a higher standard deviation of speed (SDSP), indicating an increase in the maximum velocity peaks and a decrease in the minimum velocity peaks, resulting in invariance of the mean speed. In fact, authors do agree about the increased speed variability following alcohol consumption^23,48^, demonstrating that this is a more reliable variable than mean speed for assessing driving ability.

The SDωSW was also higher in the three scenarios after alcohol use, particularly on the two-lane mountain road and in the inner-city. Although there is little information on the effects of alcohol on steering behavior, our results are in line with those of Li and colleagues^21^, who reported that steering behavior is impaired by alcohol consumption, especially when driving on curves. On the two-lane mountain road, we found a significant increase in SDωSW for both alcohol-intake conditions in the high-BrAC group, but also for the high-alcohol intake in the low-BrAC group. In addition, on the dual carriageway, as well as in the inner city, the SDωSW was significantly higher after the high alcohol intake (aAC2 condition) for the high-BrAC group. Likewise, the drivers found it more difficult to maintain the position of the car in the lane, as indicated by the increased SDLP and NTVOS. The impairments observed in the use of the steering wheel and the SDLP were slightly higher in relation to the other variables, indicating that these variables may be more sensitive. In this sense, some authors have concluded that it is more difficult to control the position of the car under the influence of alcohol, with SDLP being the variable that best assesses driving performance^22,48,49^. Response time was also significantly higher after alcohol intake, as evidenced in other work^24,50^. However, it has also been reported that alcohol does not affect this variable, as response time may be affected by the complexity of the task^51^.

Despite the deterioration of the ODPS observed in this study following alcohol use, the correlation between the BrAC level and driving performance impairment is not clear. Contrary to other findings^22^, we found no significant correlation between the ODPS and BrAC level reached. This could be explained by the phenomenon of alcohol tolerance experienced by certain people, which masks some of the negative effects of alcohol^33^. Previous work has reported that there may be acute tolerance to the impairment effects of alcohol on motor coordination and psychomotor function^52,53^. Likewise, it has also been reported that drunk-drivers act inconspicuously when driving as a compensatory behavior in order to seem sober^54^. This way, whether an association between the BrAC level and the ODPS is observed or not, would also depend on the study participants and their ability to act to compensate for a difficult situation. Our findings have shown that driving performance, measured by means of the ODPS, was significantly worse after alcohol use (for both conditions, aAC1 and aAC2) and that the deterioration was stronger in the high alcohol-intake (aAC2) condition for both groups (Fig. 4), i.e., for participants who attained a high BrAC, in addition to those with a low BrAC (BrAC < 0.25 mg/l). These results show that BrAC levels over the legal limit of 0.25 mg/l are riskier for driving due to greater deterioration in both visual and driving performance. The majority of cases in our study with levels over 0.25 mg/l (high BrAC group), had a BrAC value of between 0.25 mg/l and 0.40 mg/l. It is expected that, for a legal limit of 0.40 mg/l, which is the second most frequent limit in the world, drivers exceeding this limit will experience increased deterioration in their visual and driving performance. However, for this legal limit, drivers with a BrAC of between 0.25 mg/l and 0.40 mg/l could legally drive, despite of the impairment of visual function and driving demonstrated in this work, resulting in riskier driving. In the EU, the most common legal limit for driving is a BrAC limit of 0.25 mg/l (a BAC, Blood Alcohol Concentration, of 0.05%). In some countries, however, this value is lower: Lithuania has a legal limit of 0.20 mg/l; Poland, Sweden and Estonia have a legal limit of 0.10 mg/l; and the Czech Republic, Hungary, Romania and Slovakia apply a legal limit of 0.00 mg/l^16^.

We also determined the degree of involvement of two important visual functions in driving performance under the effects of alcohol. The regression analysis indicated two important facts. The first was that, under the effects of alcohol, the two visual variables (binocular CS and straylight) impacted the ODPS, where the latter included all the driving variables (equal independent weight) from the three scenarios. The second was that this contribution was limited (16.1%), indicating that other visual variables or other cognitive or psychomotor aspects may also have an important influence on driving performance under these conditions. This is in accordance with other authors who observed that these visual functions impacted driving performance^4,32,55,56^. This percentage is not high but still significant, which is reasonable considering that other visual functions not evaluated in this study are also associated with driving performance, such as refractive blur^57^, useful field of vision (UFOV)^1,34,56^, 1 49 52 and dynamic visual acuity^34^. We should remark that the driving performance results were obtained in daytime driving conditions, so it would be very interesting to study what happens in night-time conditions. Some studies have demonstrated that contrast sensitivity is diminished under night-vision conditions^58^. In these light conditions, impaired mesopic vision correlates with increased glare sensitivity in drivers^59^, since straylight has been reported to be an indicator of glare sensitivity^10^. Other authors have shown diminished mesopic CS and a stronger perception of halos in older drivers, both with and without visual impairment^60^. In addition, some work has reported deteriorated night-vision performance, including intraocular scattering, after alcohol consumption^19,41^. Taking into account these results, and considering our findings, we can predict that any deterioration in visual performance would have a stronger effect on driving performance in night-time driving conditions, especially after alcohol consumption.

Although a significant correlation was found between the results of the ODPS and the two visual functions obtained in this study (Fig. 5), we found no association between the deterioration of the ODPS and the visual parameters. This may be because the driving task involves not only vision, but also sensorimotor and cognitive skills that were not within the scope of this work^5^. As these cognitive skills are also impaired by alcohol^61^, studying how the deterioration of these skills impacts the ODPS could provide a better understanding of driving performance impairment under these conditions. However, the correlations obtained between the ODPS and the visual functions studied here, as well as the deterioration seen in several driving variables and the ODPS after alcohol consumption, indicates that alcohol use has an important negative influence on both driving and visual performance, especially when considering a high alcohol intake. It is important to note that these results were obtained using a driving simulator. Although it has been reported that simulators are effective for assessing driving performance^62^, more reckless and careless behavior has been observed in simulated driving with respect to on-road driving^63^.

## Conclusions

Both visual and driving performance were impaired following alcohol consumption, especially when considering moderate or high doses, where the BrAC level is above the legal limit of 0.25 mg/l for driving. Contrast sensitivity for medium–high spatial frequencies was significantly deteriorated, as was retinal straylight following alcohol consumption, especially for high BrAC levels. For driving performance, all the variables analyzed were impaired after alcohol use, with the exception of mean speed, which remained unaltered, on average, in both groups, although higher values in the standard deviation were recorded after alcohol intake. The impairment was greatest on the two-lane mountain road, which was the most difficult scenario of the three analyzed, where the SDLP and the SDωSW were most sensitive to driving impairment. Driving performance (ODPS) strongly deteriorated after alcohol use, especially for the high alcohol intake and for those participants who attained a BrAC higher than 0.25 mg/l. However, no correlation was found between the ODPS and the BrAC level reached. Our findings indicate that visual functions such as contrast sensitivity and retinal forward scattering, also have an important influence on driving performance under the effects of alcohol, especially for high alcohol intakes. For this reason, it would be interesting to consider other visual parameters, such as those studied here, in visual driving examinations, to more precisely evaluate visual function under different conditions.

## Data Availability

Available from the corresponding author on reasonable request.
